# Specific behavioral and cellular adaptations induced by chronic morphine are reduced by dietary omega-3 polyunsaturated fatty acids

**DOI:** 10.1371/journal.pone.0175090

**Published:** 2017-04-05

**Authors:** Joshua Hakimian, Ani Minasyan, Lily Zhe-Ying, Mariana Loureiro, Austin Beltrand, Camille Johnston, Alexander Vorperian, Nicole Romaneschi, Waleed Atallah, Fernando Gomez-Pinilla, Wendy Walwyn

**Affiliations:** 1 Department of Psychiatry and Biobehavioral Sciences, University of California Los Angeles, Los Angeles, California; 2 Brain Research Institute, University of California Los Angeles, Los Angeles, California; 3 UCLA Integrative Biology and Physiology, University of California Los Angeles, Los Angeles, California; Tokai University, JAPAN

## Abstract

Opiates, one of the oldest known drugs, are the benchmark for treating pain. Regular opioid exposure also induces euphoria making these compounds addictive and often misused, as shown by the current epidemic of opioid abuse and overdose mortalities. In addition to the effect of opioids on their cognate receptors and signaling cascades, these compounds also induce multiple adaptations at cellular and behavioral levels. As omega-3 polyunsaturated fatty acids (n-3 PUFAs) play a ubiquitous role in behavioral and cellular processes, we proposed that supplemental n-3 PUFAs, enriched in docosahexanoic acid (DHA), could offset these adaptations following chronic opioid exposure. We used an 8 week regimen of n-3 PUFA supplementation followed by 8 days of morphine in the presence of this diet. We first assessed the effect of morphine in different behavioral measures and found that morphine increased anxiety and reduced wheel-running behavior. These effects were reduced by dietary n-3 PUFAs without affecting morphine-induced analgesia or hyperlocomotion, known effects of this opiate acting at mu opioid receptors. At the cellular level we found that morphine reduced striatal DHA content and that this was reversed by supplemental n-3 PUFAs. Chronic morphine also increased glutamatergic plasticity and the proportion of Grin2B-NMDARs in striatal projection neurons. This effect was similarly reversed by supplemental n-3 PUFAs. Gene analysis showed that supplemental PUFAs offset the effect of morphine on genes found in neurons of the dopamine receptor 2 (D2)-enriched indirect pathway but not of genes found in dopamine receptor 1(D1)-enriched direct-pathway neurons. Analysis of the D2 striatal connectome by a retrogradely transported pseudorabies virus showed that n-3 PUFA supplementation reversed the effect of chronic morphine on the innervation of D2 neurons by the dorsomedial prefontal and piriform cortices. Together these changes outline specific behavioral and cellular effects of morphine that can be reduced or reversed by dietary n-3 PUFAs.

## Introduction

Opioids are prescribed for pain relief but are also used to induce euphoria contributing to the increasing diversion of these readily available pharmaceutical compounds for non-medical use [1, 2]. In the last decade there has been an exponential rise in the abuse of prescription opioids which have become a gateway to heroin and fentanyl abuse [3]. Together these, and other synthetic opioids have led to a meteoric rise in overdose mortalities [2–8] making the development and use of suitable interventions to address this epidemic a priority of state and federal regulators [9, 10].

The initial rewarding effect of opioids creates a positive reinforcing stimulus that drives further opioid exposure to obtain the same euphoria. However, as opioid use continues and allostatic adaptations within reward and non-reward circuits occur, the drive to obtain further opioids changes to negative reinforcement. This type of reinforcement increases opioid-seeking behavior to escape the dysphoria between each drug exposure [11–13]. Coupled with opioid tolerance and dependence, symptoms typical of chronic opioid exposure, this leads to a state of negative affect with associated symptoms of anxiety and depression [14, 15].

Docosahexanoic acid (DHA) is an essential poly-unsaturated long chain fatty acid (PUFA; c22:6n-3) that is obtained from dietary sources, mostly from deep-sea fish that require long chain fatty acids to maintain membrane fluidity in cold temperatures. DHA is required for development [16] and is enriched in mammalian brains, particularly the grey matter where it is a structural component of plasma, microsomal and synaptic membranes [17, 18]. DHA is also involved in diverse cellular functions through recently identified receptors [19–23]. The beneficial effects of supplementary DHA for many conditions and diseases have been the subject of ongoing research. Of these, the possibility that dietary omega-3 supplementation relieves the anxiety and depression of many comorbid disorders is a recurring theme (reviews; [24–26]). Accordingly n-3 supplements enriched in DHA have been shown to reduce anxiety in preclinical [24, 27, 28] and clinical trials [25, 29–32].

We propose that chronic morphine will induce adaptations at the behavioral, cellular and circuitry levels that dietary n-3 PUFAs, enriched in DHA, will reduce. Furthermore, it is possible that the allostatic adaptations that are offset by supplemental n-3PUFAs may not be part of the known signaling cascades of this opiate. The objective of this study is to define the effect of morphine and dietary n-3 PUFAs on specific behavioral and cellular measures in a mouse model of chronic opioid exposure. We have used a range of techniques to define this interaction and conclude that n-3PUFAs may be beneficial in offsetting specific adaptations induced by chronic morphine.

## Materials and methods

### Animals

All the experiments were conducted in accordance with the AALAC Guide for the Care and Use of Laboratory Animals and approved by the UCLA IACUC committee. Wildtype C57BL/6J male (n = 55) or female (n = 8) mice were used for all experiments except the viral tracing experiment in which male C57BL/6J Drd2-cre (MMRC 032108-UCD, n = 17) mice were used.

### Dietary and opioid interventions

Animals, 6–8 weeks of age at the start of the experiment, were maintained on a control lab chow diet containing 0.5% DHA (Control) alone or supplemented with 2.5% DHA, 1.1% EPA and 0.75% other omega-3 PUFAs, (Nordic Naturals, Watsonville, CA) for 8 weeks. We used a twice daily ascending (TDA) schedule of morphine injections over 8 days. This consisted of two injections 10-12h apart of 10, 20, 30, 40 mg/kg (10μl/g body weight) on days 1, 2, 3, 4 respectively followed by 50mg/kg on days 5, 6, 7, 8. Mice receiving saline underwent the same schedule of injections but saline (10μl/g body weight) was injected.

### Behavioral tests

A battery of behavioral test were used to examine the effect of chronic morphine and n-3 PUFA supplementation. The data from all behavioral tests were analyzed using Prism (v6.0) with significance accepted at p<0.05.

#### i. Wheel-running

Spontaneous wheel running activity was measured in 14 mice (8 males and 6 females) after 8 weeks of DHA or control diet. Morphine or saline was injected by the TDA schedule and overnight wheel running activity assessed on days 1, 4 and 8. Statistical Analysis: The data were analyzed by one-way ANOVA with repeated measures and the Holm-Sidak post-hoc test to assess differences in the total distance run, and two-way ANOVA and the Holm-Sidak post-hoc test to assess the interaction of time (5’ bins) and intervention (control or DHA diets).

#### ii. Elevated Plus Maze (EPM)

Five hours after the last morphine injection, mice were placed in the central zone of the EPM; 21” height, 26” arm length, 3” arm width, at 10 lux, n = 6 male mice/group. Statistical Analysis: Behavior was video-tracked by an infrared camera and Ethovision (Noldus XT8.0) and time spent and number of entries in the outer half of the open arms assessed. The data were analyzed by 2-way ANOVA and the Holm-Sidak post-hoc test to assess the interaction of time (5’ bins) and intervention (control or DHA diets).

#### iii. Thermal analgesia

The analgesic effect of a single dose of morphine was assessed by the response to tail-immersion in warm water (49.5°C) in mice on the DHA or control diet for 8 weeks, n = 5 males/group. After a basal measure was taken, morphine (10mg/kg) was injected subcutaneously and, 30’ later, the time taken (s) to shake or remove the tail from the water measured. Statistical Analysis: The data were analyzed by the Student’s t-test to assess the effect of diet on baseline pain responses and by 2-way ANOVA and the Holm-Sidak post-hoc test to assess the effect of treatment and intervention (control or DHA diets).

#### iv. Locomotion

The initial locomotor effect of morphine and sensitization of this response was assessed after the first morphine injection of the day on days 1, 4 and 8 of the morphine injection protocol. Mice from DHA or control groups were initially placed in open field chambers (10.5x10.5”, 10 lux) for 15’, then injected with morphine or saline, the locomotor response assessed for the following 60’ and video-tracked by Ethovision, 5 male mice were used for each of the 4 groups. Statistical Analysis: The data were analyzed by 2-way ANOVA and the Holm-Sidak post-hoc test to assess the interaction of time (day) and intervention (control or DHA diets).

### Gas chromatography

Total lipids from the striatum and frontal cortex from 5 male and 3 female mice were extracted after 8 weeks of the DHA diet according to the protocol by Bligh and Dyer [33]. Briefly, the tissues were homogenized with chloroform-methanol (2∶1 vol∶vol) including 0.005% butylated hydroxytoluene and tricosanoic acid methylester as an internal control. After centrifugation, the liquid was mixed with 0.9% NaCl and the chloroform layer collected and dried under nitrogen. Lipids were transmethylated (90°C for 1h) using 14 wt/v% Boron Trifluoride/methanol. Fatty acid composition was analyzed by gas chromatography (Clarus 500, PerkinElmer, Waltham, MA) equipped with an Elite-WAX column (PerkinElmer) with an injector and detector temperature of 250°C and 300°C respectively. Hydrogen was used as the carrier gas with a split ratio of 100∶1. Statistical analysis: Identified peaks were compared with standards (GLC Reference standard 682, Nu-Chek-Prep, Elysian, MN, and 37-component FAME mix, Sigma-Aldrich, Carlsbad) and data analyzed by 1-way ANOVA with post-hoc Tukey tests (Prizm v6).

### qPCR

Total RNA was isolated from 1mm striatal punches pooled from both hemispheres (Direct-zol MiniPrep kit, ZYMO Research, Irvine, CA). Total RNA (100 ng) was converted to cDNA (qScript^™^ cDNA Synthesis, Quanta Bioscience, Beverley, MA) followed by Sybr-green based quantitative PCR, (PerfeCta SYBR Green FastMix kit; Quanta Bioscience) and 40 cycles of 15-30s at 95°C and 60°C (CFX96 Real-Time PCR Detection System, Bio-Rad). Pre-designed primers (Kiqstart, Sigma Aldrich) were used to assess the expression of; dopamine receptor 1 (D1), dopamine receptor 2 (D2), prodynorphin (Dyn) and preproenkephalin (Eenk) and the endogenous housekeeping gene, glyceraldeyde 3-phosphate dehydrogenase (GAPDH). Statistical analysis. Data from 6 and 7 male mice were collected for each of the 2 control groups, saline and morphine respectively, and 6 male mice for each of the DHA groups; saline or morphine. Data were analyzed by the ΔΔCt method where Ct is the cycle at which fluorescence first increased above background. The ΔCt value was calculated as the difference between the Ct value of each sample from GAPDH and the ΔΔCt as the difference between the experimental and control samples. The date are expressed as the 2^-ΔΔCt and analyzed by 1-way ANOVA with Holm-Sidak post-hoc tests (Prism v6).

### Electrophysiology

#### i. Slice preparation

Twenty-four hours after the last morphine or saline injection, mice were euthanized by isoflurane, the brain rapidly extracted and immersed in an oxygenated, ice cold, solution containing (in mM); 140 K-gluconate, 15 Na^+^ gluconate, 4 NaCl, 10 HEPES, 0.2 EGTA, pH 7.2, 209–310 mOsm. 300μm coronal slices were cut on a VT1000S vibratome (Leica Microsystems, Wetzlar, Germany), incubated in artificial cerebrospinal fluid (ACSF, in mM: 130 NaCl, 26 NaHCO_3_, 3 KCl, 2 MgCl_2_, 1.25 NaHPO_4_, 2 CaCl_2_, and 10 glucose^,^ pH: 7.4, osmolality: 300–310 mOsm), and perfused with 95% O_2_-5% CO_2_ at RT for at least 1h before recording.

#### ii. Electrophysiological recordings

A Slicescope (Scientifica, UK) consisting of an upright, modified Olympus BX51W1 microscope, manipulators and controllers coupled with an Axopatch 200B amplifier, NidAQ digitizer and winEDR (University of Stratchlyde, Glasgow, Scotland) software were used. Borosilicate glass capillaries (World Precision Instruments, Sarasota, FL) were pulled using a micropipette puller (P-97, Sutter Instruments Company, Novato, CA) to a resistance of 3–4 MΩ when filled with intracellular solution (in mM; 125 Cs-methanesulfonate, 3 KCl, 4 NaCl, 1 MgCl_2_, 5 Mg ATP, 9 EGTA, 8 HEPES, 1 GTP Tris, 10 phosphocreatine disodium and 0.1 leupeptin, pH 7.25–7.3, osmolality, 280–290 mOsm). Evoked EPSCs (eEPSCs) were recorded from MSNs in the NAc shell (NAcsh) in the presence of the GABA_A_ receptor antagonist, bicuculline (BIC, 10 μM) in the external solution, while holding the membrane potential at -70 mV or +40 mV, to obtain AMPA and NMDA currents respectively. The slices were perfused with 1–2 ml/min, oxygenated ACSF at RT. The AMPA and NMDA antagonists, 6-cyano-7-nitroquinoxaline-2, 3-dione (CNQX, 10 μM) and amino-5-phosphonovaleric acid, (AP-5, 50 μM) respectively were added into the external solution as needed. Ro25-60981 (1mM, Tocris) was also used to block Grin2B-NMDA currents. MSNs within the NAcsh were visualized by infrared, differential interference microscopy and identified by their size (8-12micron), positive reversal potential and basic membrane properties. A concentric bipolar electrode (FH, Bowdoinham, ME) was placed at the same plane 50-100microns from the recorded cell. The slices were stimulated with a ~500mA current at a frequency of 0.3Hz and adjusted to obtain a submaximal current of~500pA current at -70mV. All antagonists were perfused for 300s at -70mV before the 60s stimulation protocol was applied. Statistical analysis: Two—4 recordings were obtained from 4 male mice for each of the 4 groups, and the data analyzed using WinEDR and WinWCP software. Data are represented as a ratio, or as a percentage of the peak amplitude current, and were analyzed by one-way ANOVA and Holm-Sidak’s post-hoc test. Recordings in which the series resistance was more than 25 MOhms initially or drifted by more than 20% over time were excluded. Basal membrane properties and stimulating currents were not different across groups and are shown in S1 Table.

### Viral tracing

A retrogradely-transported pseudorabies virus (PRV263) containing the Brainbow 1.0L cassette [34, 35], (0.56 μl; 2.5 x 10^5^ pfu) was injected into the right striatum (x:+/-2.0, y:+0.7, z: -3.5mm) of transgenic mice expressing Cre recombinase in D2 neurons [36]. These mice had been on a control or DHA-enriched diet. After 48h, the brains were extracted, submerged in 4% paraformaldehyde overnight followed by 30% sucrose for 48h and then frozen. Alternate 50micron section were processed for GFP immunohistochemistry, mounted in Prolong with DAPI (Thermofischer, Carlsbad, CA) and imaged by a slide scanner (Aperio Versa 200 Digital Pathology Scanner, Leica Biosystems). Each section was registered to the Allen Brain Atlas and the number of labeled cells counted by a program written in python by the lab, provided in S2 Table. Statistical analysis: Three-6 brains were assessed per condition and analyzed by 2-way ANOVA and Tukey’s post-hoc test for 69 primary brain regions listed in S3 Table.

## Results

### i. DHA offsets the anxiety induced by chronic morphine but does not alter morphine-induced analgesia or locomotion

The protocol of chronic morphine used in this study is known to induce anxiety [37], and as DHA supplementation may reduce this symptom of negative affect [25], we proposed that dietary DHA would reduce the increase in anxiety associated with chronic morphine exposure. We used 2 measures of anxiety; 1) wheel running, a form of environmental enrichment that reduces the anxiolytic profile of morphine dependence [37] and, 2) the elevated plus maze (EPM), a well-known measure of anxiety.

### ii. Wheel running

**Fig 1:**
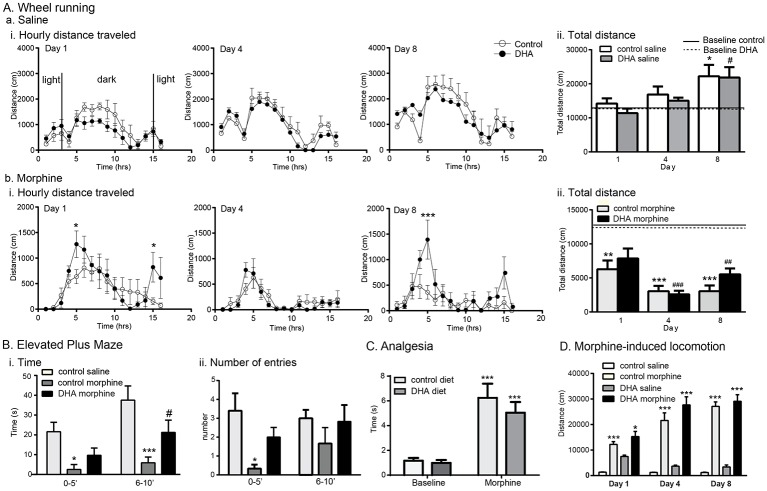
DHA supplementation attenuates the anxiety induced by morphine without altering analgesia or locomotion, established effects of this opiate. **A. Wheel running** activity was measured over 16h; 3h of light, 12h of dark, 1h of light. The distance run (cm) every hour is shown in the 3 left panels and the total distance run in the column graphs on the right. DHA supplementation did not alter wheel running in male mice receiving saline (Ai) which by day 8 had increased their activity compared with day 1. *, # p<0.05 vs pre-saline injection activity, shown in the solid (control diet) or dashed (DHA group) lines, of the same treatment. B. Total distance run, depicted in the column graphs, shows that morphine reduced wheel running in both DHA and control groups when compared with pre-treatment levels; **, *** p<0.05 and 0.005 respectively vs basal activity in the control group. ##, ### p<0.05 and 0.005 respectively vs basal activity in the DHA group. However, the hourly time bin data show that DHA attenuated this reduction at specific time-points, 5 and 15 h post-morphine injection on day 1, and 5 hours on day 8, *p<0.05, ***p<0.001 vs control morphine at the same timepoint. **B. Elevated Plus Maze (EPM).** The time spent and frequency of entry into the second, outer half of the EPM was assessed 5h following the last injection of the 8^th^ day of the morphine or saline injection protocol. Mice on the control diet receiving morphine spent less time and entered less into this region than control mice receiving saline. Supplemental DHA partially reversed this profile. *, ***p<0.05 and 0.001 respectively vs the control saline group, #p<0.05 vs control saline and control morphine. **C. Morphine-induced analgesia.** The effect of DHA on morphine-induced thermal analgesia was assessed by the tail immersion test at 49.5°C. There was no effect of diet on basal tail-flick latency. Morphine (10mg/kg s.c.) delayed this response in both control and DHA groups (*** p<0.001 vs baseline measures). **D. Morphine induced locomotion.** i. This effect if morphine was assessed immediately following the first of the daily morphine injections on days 1, 4 and 8 of the TDA morphine injection protocol. The total distance traveled showed no effect of DHA on either the initial locomotor response, or subsequent, sensitized responses, which increased above saline-treated mice on the matching diet, *p<0.05, ***p<0.001 vs saline of the matching control or DHA diet.

Males: DHA did not alter basal wheel running activity in untreated control and DHA groups (control: 13115±1240cm, DHA: 12082±42cm F_(1,3)_ = 0.9, p = 0.4). Whereas saline mice increased wheel running activity, morphine inhibited wheel running (F_(13,69)_ = 19.1, p<0.0001,pre-training control morphine day 8 vs l; p = 0.0001, pre-training control vs saline day 8; p = 0.008). DHA modified, but did not reverse the effects of morphine and increased wheel running at specific time bins, 5 and 15h after the morphine injection on day 1 (5h; control: 652±158cm, DHA: 1272±260cm, and 15h; control: 150±76cm, DHA: 823±292cm; p<0.05) and at 5h on day 8 (5h; control: 311 ±143cm, DHA:1392±382cm, p<0.001, Fig 1A). Females: Similar to males, morphine decreased wheel-running activity (F_(7,28)_ = 6.7, p = 0.<0.001), but this was not altered by supplemental DHA (F_(15,129)_ = 1.5, p = 0.1, S1 Fig). As female mice did not show an effect of DHA supplementation, males were used in all subsequent experiments unless stated otherwise.

### iii. EPM

We targeted the time window, 5h after morphine, when DHA increased wheel running activity in morphine-treated mice. Mice that had been on the DHA or control diet for 8 weeks followed by the TDA morphine injection protocol were placed on the EPM for 10’ and data analyzed in 5’ bins. We found a significant effect of treatment on time spent in the outer half of the open arms (F_(2,15)_ = 17.4, p = 0.0001, Fig 1Bi). Compared with control mice, those receiving morphine spent less time in this region during both time bins (0–5’; control saline: 21.6±4.8s, control morphine: 2.5±2.5s, p<0.05 and 6–10’: control saline: 37.5±7.2s, control morphine: 5.9±2.9s, p<0.001). Supplemental DHA partially reversed this behavior: Time spent in this region during the first 5’ bin by the DHA-morphine mice (9.7±3.7s) was not different from either the control saline or control morphine groups. However, during the second 5’ bin, the DHA morphine mice spent more time in this region than control morphine mice (control morphine: 5.9±2.9s, DHA morphine: 21.2±6.2s, p<0.05), although this was less than control saline mice (37.5±7.2s, p<0.05). There was also an effect of treatment on the number of entries into the second half of the open arms (F_(2,15)_ = 5.1, p = 0.02, Fig 1Bii). During the first 5’ bin, control morphine mice entered this region less than control saline mice (control morphine: 0.3±0.2, control saline: 3.4±0.9, p<0.01). However, the number of entries by the morphine-DHA mice (2.0±0.5) did not differ from those of either control saline or control morphine mice. There was no effect of treatment during the second 5’ bin. We next assessed the effect of DHA on known effects of morphine; thermal analgesia and locomotion.

### iv. Thermal analgesia

There was no effect of diet on basal nociception (control diet: 1.2±0.2s, DHA diet: 1.0±0.2s, t = 0.6, df = 8, p = 0.6). A single morphine injection delayed the response to heat (control diet: 6.2±1.1s, DHA diet: 5.0±0.9s, F_(1,4)_ = 30.9, p = 0.005) but there was no effect of diet (F_(1,4)_ = 3.1, p = 0.2, Fig 1C).

### v. Morphine-induced locomotion

The locomotor effect of morphine was assessed during the TDA schedule; after the1^st^ injection on day 1 (10mg/kg), the 6^th^ injection on day 4 (30mg/kg) and the final injection on day 8, (50mg/kg). After 15’ of basal locomotion, morphine was injected and locomotor distance measured over 60’. Morphine induced locomotion in both control and DHA groups (Day 1; control saline: 1353±103cm, control morphine: 12216±1109cm, DHA saline: 7472±545cm, DHA morphine: 15278±2042cm, F_(3,16)_ = 8.6, p<0.001. Day 4; control saline; 1250±72cm, control morphine; 21582±2998cm, DHA saline; 3692±383cm, DHA morphine; 27693±3187cm, F_(3,1)_ = 26.0, p<0.001. Day 8; control saline; 1224±125cm, control morphine; 27129±1760cm, DHA saline; 3381±779cm, DHA morphine; 29101±2580cm, F_(3,12)_ = 75.7, p<0.001). However, there was no effect of DHA on the total locomotion for each of these days (F_(2,8)_ = 0.4, p = 0.7, Fig 1D). There was also no effect of diet on the sensitization of this response over time (F_(2,8)_ = 0.4).Together these data suggest that a DHA-enriched diet partly offsets the anxiogenic effects of chronic morphine but does not alter basal thermal pain or the ability of morphine to induce analgesia or hyperlocomotion.

### Region-specific effects of dietary DHA and morphine on brain DHA content

As DHA supplementation altered specific behaviors induced by morphine, we next examined whether the supplemental DHA protocol used altered DHA content of the frontal cortex and striatum, regions known to be affected by prior opioid exposure, the frontal cortex and striatum [38].

#### i. Frontal cortex

**Fig 2:**
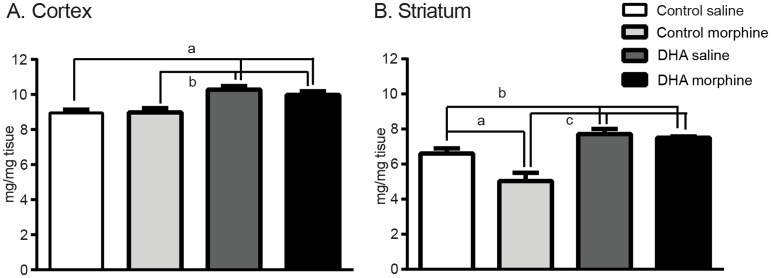
Region-specific effects of dietary DHA and morphine on brain DHA content. **A. Frontal cortex.** DHA supplementation increased DHA content following saline or morphine treatment with no further effect of morphine. a and b; p<0.05. **B. Striatum**. Chronic morphine decreased DHA tissue content and DHA supplementation increased DHA content following saline or morphine. a; p<0.01, b; p<0.05. c; p<0.0001.

Supplemental DHA increased DHA content following morphine or saline treatments (control saline: 8.9±0.3mg/mg, control morphine: 8.8±0.3mg/mg, DHA saline: 10.4±0.3mg/mg, DHA morphine: 10.3±0.2mg/mg, F_(3,28)_ = 10.4, p<0.001, Fig 2A) However, morphine had no effect on cortical DHA content in control (p = 0.9) or DHA (p = 0.3) treated mice (Fig 2A). As female mice did not show an effect of DHA supplementation (F_(3,8)_ = 2.1, p = 0.2 S2 Fig), males were used in all subsequent experiments.

#### ii. Striatum

In contrast to the cortex, the striatum showed an effect of both DHA and morphine on striatal DHA content (control saline: 6.6±0.3mg/mg, control morphine: 5.0±05mg/mg, DHA saline: 7.7±0.3mg/mg, DHA morphine: 7.5±0.07mg/mg, F_(3,14)_ = 17.8, p<0.001, Fig 2B). Morphine decreased DHA content in control tissue (control saline vs control morphine; p<0.001), but did not change DHA content in tissue from DHA mice (control morphine vs DHA morphine, p = 0.6). Supplemental DHA increased DHA content in both morphine and saline treated mice (p<0.05). Together these results show that morphine reduced DHA content in striatal tissue and DHA supplementation, in increasing DHA content, offset this reduction. Based on these findings we focused on the striatum in subsequent assays.

### Dietary DHA reverses the effects of morphine on specific striatal glutamate receptor subunit expression

As morphine or DHA may independently alter glutamatergic receptor expression and function [39, 40], we first used qPCR to assess the effect of morphine and DHA on the expression of specific glutamatergic subunits.

#### i. Gria1 (GluR1)

**Fig 3:**
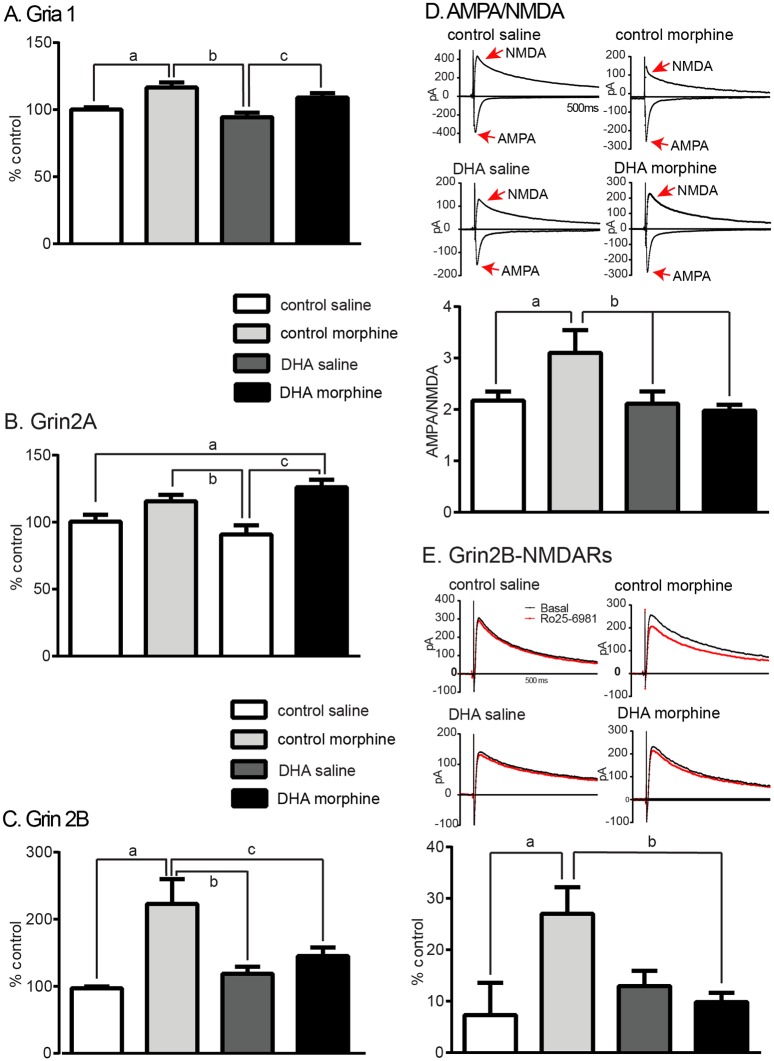
Dietary DHA reverses the effects of morphine on specific striatal glutamate receptor subunit expression and function. **A. Gria 1.** DHA supplementation did not alter the morphine-induced increase in Gria1 transcript levels. a; p<0.01, b; p<0.001, c: p<0.05 **B. Grin 2A.** DHA supplementation did not alter the effect of morphine on Grin2A expression which increased following morphine in control or DHA groups. a and b; p<0.05, c; p<0.01. **C. Grin 2B** DHA supplementation reduced the morphine-induced increase in Grin2B expression. a; p<0.0001, b; p<0.01, c; p<0.005. **D. AMPA/NMDA ratio** This index of glutamatergic signaling, assessed at -70 and +40mV, increased following morphine. DHA reduced this increase. a and b; p<0.05. **E. Grin2B-NMDARs** NMDARs were recording in the absence and presence of a Grin2B antagonist, Ro25-6981 (1μM). Morphine increased Grin2B-containing NMDARs and this was reduced following DHA, a; p<0.01, b; p<0.01.

We found a significant treatment effect on Gria 1 expression (control saline: 100±2%, control morphine: 117±4%, DHA saline: 94±3%, DHA morphine: 109±3%, F_(3,16)_ = 9.8 p<0.001, Fig 3A). Morphine increased Gria1 transcript levels following both control (control saline vs control morphine p<0.005) or DHA diets (DHA saline vs DHA morphine, p<0.05). However, there was no interaction of diet and morphine (control morphine vs DHA morphine, p = 0.2).

#### ii. Grin2A (NR2A)

Morphine and DHA showed a significant treatment effect on Grin2A transcript levels (control saline: 100±5%, control morphine: 115±5%, DHA saline: 91±7%, DHA morphine: 126±7%, F_(3.16)_ = 7.5, p = 0.002, Fig 3B); morphine increased Grin2A expression following the DHA diet (p<0.005) and trended towards an increase following the control diet (p = 0.08). However, there was no effect of supplemental DHA on this transcript (control saline vs DHA saline, p = 0.3).

#### iii. Grin2B (NR2B)

Grin2B transcripts showed a significant interaction (control saline: 97±3%, control morphine: 223±37%, DHA saline: 119±10%, DHA morphine: 145±13%, F_(3, 20)_ = 9.3, p<0.001, Fig 3C). Morphine increased Grin2B expression (control saline vs control morphine; p<0.0001) but this did not occur following DHA (DHA saline vs DHA morphine; p = 0.12). In summary, chronic morphine increased the expression of the 3 glutamatergic subunits examined but DHA offset the effect of morphine on the expression of Grin2B, but not Gria1 or Grin2A.

### Supplemental DHA reduces the effect of morphine on glutamatergic plasticity

Chronic morphine has been shown to increase the AMPA/NMDA ratio and the contribution of NR2B subunits to the NMDA current in the NAcsh [41]. We therefore used slice electrophysiology *ex vivo* to assess evoked EPSCs and measured the AMPA/NMDA ratio in medium spiny neurons (MSNs) in the N.Acsh. We found that chronic morphine increased this ratio (control saline: 2.17±0.2, control morphine: 3.2±0.4, DHA saline: 2.1±0.2, DHA morphine: 2.0±0.1, F_(3,43)_ = 3.9, p<0.01, Fig 3D). DHA supplementation reduced this increase to basal levels (p = 0.9, DHA morphine vs control saline). Chronic morphine also increased the contribution of Grin2B-NMDARs to NMDAR currents (control saline: 7.3±6.3%, control morphine: 27.0±5.2%, DHA saline: 13.0±3.0%, DHA morphine: 9.8±1.8%, F_(3,28)_ = 3.8, p<0.05, Fig 3E), an effect that was reduced by DHA (DHA morphine vs control saline; p = 0.7). There was no effect of DHA on either the AMPA/NMDA ratio or on the proportion of Grin2B-containingNMDA currents in saline treated mice (p = 0.7 and p = 0.4 control saline vs DHA saline respectively). In summary DHA reversed the effect of morphine on the AMPA/NMDA ratio and on Grin2B-NMDARs in striatal MSNs.

### DHA offsets the effect of morphine in specific striatal neuronal subtypes

**Fig 4:**
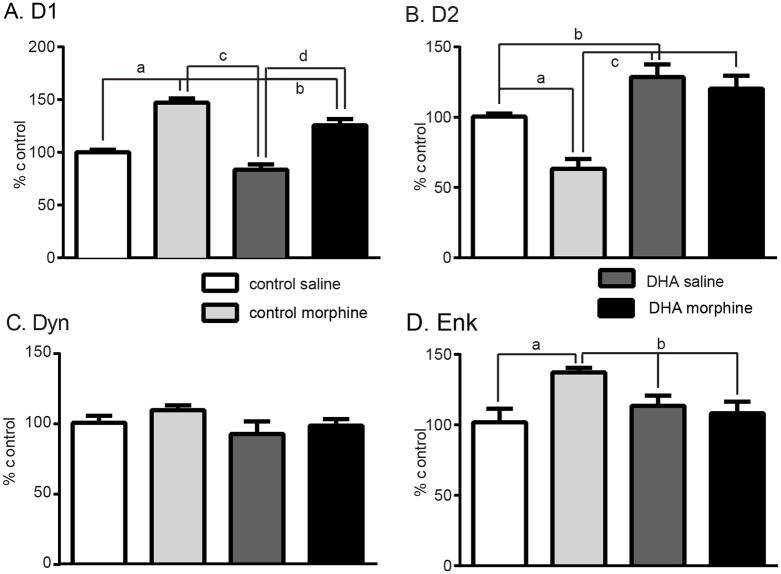
The expression of genes enriched in D2-, but not D1-, enriched striatal neurons are altered by both supplemental DHA and morphine. **A. D1** DHA supplementation did not alter the morphine-induced increase in D1 transcript levels. a, c, d; p<0.001, b; p< 0.05. **B. Dyn** Neither morphine nor DHA altered the expression of Dyn, a gene enriched in D1-neurons. **C. Drd2** Morphine reduced D2 expression while DHA supplementation increased D2 expression following saline or morphine, a; p<0.0, b; p<0.05, c; p<0.001.**D. Enk** Morphine increased the expression of Enk, a D2-enriched gene. This was reduced following DHA in either morphine or saline treated mice. a; p<0.01, b; p<0.05.

MSNs are either enriched in dopamine 1 (D1) or dopamine 2 (D2) receptors and project to the substantia nigra; direct pathway D1-MSNs, or globus pallidus; indirect pathway D2-MSNs. D1-MSNs have been implicated in the initial response to drugs of abuse whereas D2-MSNs are often associated with the development of dependence, withdrawal and aversion [42–45]. So as to further determine the striatal neuronal profile affected by morphine in these neuronal subtypes, we assessed the expression of D1-enriched genes; D1 and Dyn, and D2-enriched genes; D2 and Enk. Morphine increased D1 expression (control saline: 100±2%, control morphine: 147±4%, DHA saline: 84±5.%, DHA morphine:126 ±6%, F_(3,22)_ = 42.1, p<0.001, Fig 4A), and, although DHA did reduce this increase (p<0.01), expression remained higher in DHA-morphine compared with DHA-saline (p<01) or control saline (p<0.001) tissue. In contrast, D2 transcript levels showed a drug and treatment effect, (F_(3,21)_ = 16.0, p<0.0001, Fig 4B). Morphine reduced D2 expression in control tissue (control saline: 100±2%, control morphine: 63±7%, p<0.01), an effect that was not seen in DHA tissue (DHA saline: 129±9%, DHA morphine: 120 ±9%, p = 0.5). DHA also increased D2 expression in control tissue (control saline vs DHA saline p<0.05). Morphine did not alter the expression of Dyn, (control saline: 101±5%, control morphine: 110±3%, F_(3.16)_ = 1.4, p = 0.2, Fig 4C) but increased that of Enk (control saline: 101±10%, control morphine: 137±3%, F_(3,16)_ = 4.3, p = 0.004, Fig 4D). DHA had no effect on Dyn expression (DHA saline: 93±9%, DHA morphine: 99 ±5%, p = 0.2, Fig 4C) but reduced the morphine-induced increase in Enk expression (DHA saline: 113±7%, DHA morphine:108 ±8%, p = 0.0, Fig 4D). In summary morphine altered the expression of genes in both D1 and D2 neurons but supplemental DHA offset this effect in D2 neurons.

### Polysynaptic viral tracing shows an effect of DHA and morphine on striatal innervation

**Fig 5:**
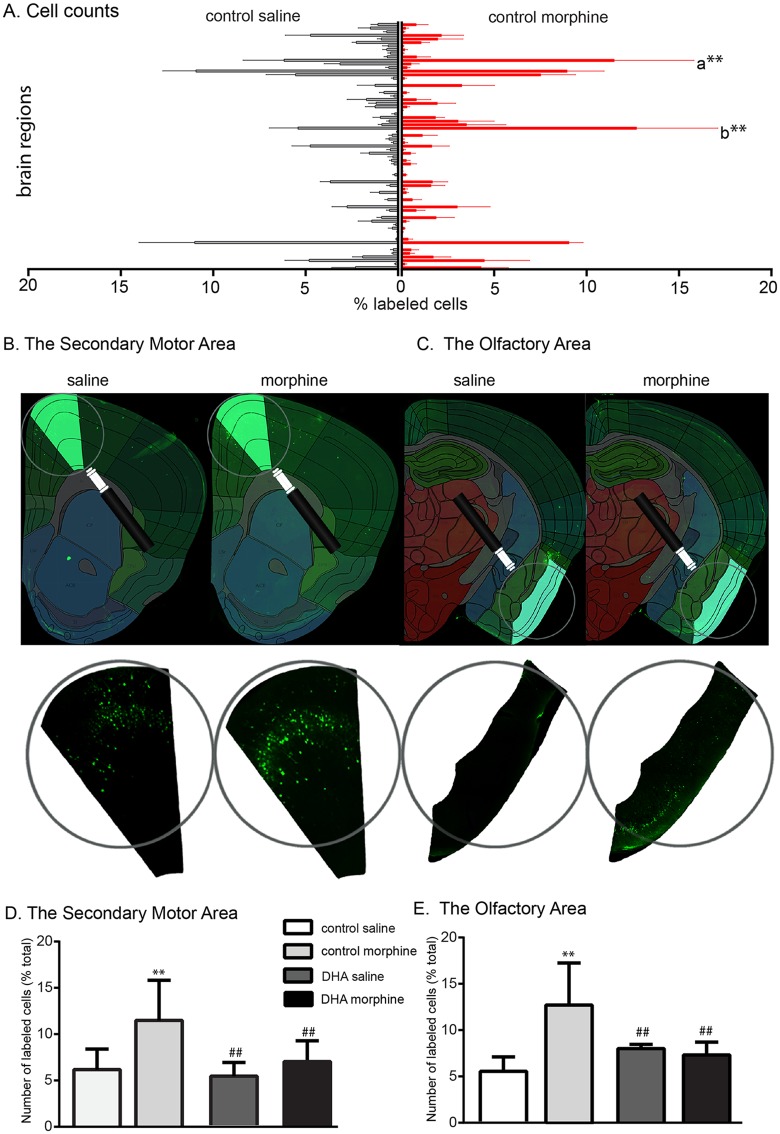
Supplemental DHA reverses the effect of morphine on the D2 striatal connectome. Polysynaptic pseudorabies viral tracing and automated cell counting was used to define the effect of morphine and DHA on the striatal connectome. Cells in 69 primary brain regions were counted, normalized to the total number of labeled cells and data from control morphine and saline mice shown in **A**. Two regions showed an effect of morphine and DHA; the secondary motor (a) and the olfactory (b) areas. **B, C**. An example of the labeling and registration to the Allen Brain Atlas of these two areas. **D, E.** Morphine increased secondary motor- and olfactory area-striatal connectivity. This was reversed by the DHA diet. **p<001 vs control saline, ##p<0.01 vs control morphine.

Chronic drug use has been shown to have a drug- and cell-specific effect on dendritic spine density and branching [46–52]. This structural plasticity could affect the functional connectivity of the striatum which DHA, as a structural component of the membrane [53], may influence. To examine this we used a retrogradely transported polysynaptic pseudorabies virus containing the floxed Brainbow cassette expressing tdTomato as the default fluorescent marker [35, 54]. When exposed to Cre recombinase, sections of the Brainbow cassette between the loxP sites are deleted and the virus and subsequent virions express yellow fluorescent protein. As our gene expression data showed that morphine altered the profile of D2, but not D1, MSNs, we used D2-cre mice for these experiments and euthanized the mice after 48h when the virus would have crossed 2–3 synapses [34]. We used an automated registration and cell counting program (S3 Table) and counted the number of labeled cells in every other section of the brain, plates 113–360 of the Allen Brain Atlas (http://www.brain-map.org/). We controlled for variance in the total number of labeled cells between mice by expressing the data as a percent of the total cell count (Fig 5A). Two-way ANOVA analysis of the 69 principal regions (F_(3, 893)_ = 0.1) highlighted 2 areas; a). The secondary motor area: Morphine increased the number of labeled cells in this region (control saline: 6.1±2.2%, control morphine: 11.5±4.3%, p<0.01) and DHA reduced this increase (control morphine vs DHA morphine (6.9±2.2%), p<0.01, Fig 5B and 5D). b). A similar effect was seen in the olfactory area which showed an effect of morphine (control saline: 5.4±1.6%, control morphine: 12.7±4.4%, p<0.01) and an effect of DHA and morphine (control morphine vs DHA morphine: 7.3±1.4%, p<0.01, Fig 5C and 5E). Closer examination of the data shows that, within this primary region, the labeling was concentrated in the piriform cortex for all groups.

## Discussion

This preclinical study shows that specific behavioral and cellular effects of chronic morphine can be reduced by a diet supplemented with n-3 PUFAs. The 8 week n-3 PUFA dietary intervention used in this study reduced the increased anxiety following chronic morphine and reversed the effect of repeated morphine exposure on striatal DHA content. This dietary intervention also normalized morphine-induced glutamatergic plasticity and increased Grin2B expression and the proportion of Grin2B-NMDAR currents in the striatum. Furthermore the effect of morphine on the expression of genes enriched in D2-MSNs and on the D2-striatal connectome was offset by this DHA-enriched diet. These data show how this intervention offset allostatic adaptations known to be induced by chronic opioid exposure [38] without altering the proto-typical effects of activating the mu opioid receptor, the predominant target of morphine and other opioids.

Although this is the first study showing an effect of supplementary DHA following chronic morphine, there are several indications that DHA status may interact with drug addiction. Humans dependent on opiates or nicotine often consume a lipid-poor diet resulting in low plasma DHA levels [55, 56]. These DHA-deficient diets alter the DHA content of specific brain regions, decrease membrane fluidity and the expression of multiple pre- and post-synaptic proteins [57–60]. A similar effect may be induced by the abused drug itself; methamphetamine induces region-specific changes in brain DHA content [61, 62]. Drugs of abuse also reduce metabolic status, brain lipid microviscosity, alter dendritic spine morphology, and increase neuroinflammation [63–71], changes which supplemental DHA may reverse [72–76]. Supplementary DHA also decreases stress, anxiety and aggression, behaviors often associated with relapse [77–79].

One of the clear benefits of dietary n-3 supplementation is an inhibition of inflammation. The *in vivo* DHA biosynthetic products, the D and E resolvins, signal through specific G-protein coupled receptors to block pro-inflammatory cascades [80]. As chronic opioid exposure activates microglia [81], and supplemental DHA reverses microglial activation [82], the beneficial effects of n-3 PUFAs seen in this study may be due to a reduction in neuroinflammation.

Our data shows several specific effects, one is the regional and cellular interactions of morphine and DHA. As the striatum is a key structure mediating reward, opioids are known to alter glutamatergic function, and D2 MSNs are involved in withdrawal and dependence associated with chronic opioid exposure [41–43], the effect of morphine on excitatory transmission and the cells of this region is not surprising. The changes in striatal connectivity of the orbital area, in particular the piriform, which innervates D2 neurons and is activated by chronic morphine exposure and withdrawal [36, 83, 84], and the secondary motor area, or the dorsomedial prefrontal cortex, which is involved in the anticipation of reward and goal-directed behavior [85], further implicate D2 MSNs as an important contributor to an opioid-induced allostatic load. However, the ability of DHA to offset these changes within this circuit suggests a regional, signaling and cellular sensitivity to this PUFA. Another key feature is a gender-specific interaction of DHA and morphine. Most of our experiments were conducted in male mice but when females were included, we found little evidence of an interaction between morphine and DHA, suggesting a gender-specific effect. The underlying mechanisms accounting for these specific effects warrant further investigation.

As an integral structural component of the cell that is also required for different cellular and metabolic processes, n-3 PUFAs are essential for normal growth and development. However, the western diet of some, but not all humans, has become increasingly low in n-3 yet higher in n-6 PUFAs. This imbalance has been implicated in several developmental abnormalities [86]. For this study we chose a control diet low in DHA content so as to mimic that of typical western diets. This initial DHA status could both contribute to the cellular and behavioral effects of opioids and to the ability of dietary DHA to offset these changes.

In summary this study provides initial pre-clinical evidence that supplemental DHA reverses specific cellular and regional effects of chronic morphine. This dietary intervention also reduced the increase in anxiety, or state of negative affect, that could be a driving contributor to opioid-seeking behaviors and relapse [38]. We propose that, in addition to the current pharmaceutical compounds such as narcan or buprenorphine, that target the mu opioid receptor, DHA could be used to reduce the allostatic adaptations associated with chronic opioid exposure.

## Supporting information

S1 Fig n-3 PUFA supplementation does not alter wheel running in female mice. Wheel running activity was measured over 16h; 3h of light, 12h of dark, 1h of light. The distance run (cm) every hour is shown in the 3 left panels and the total distance run in the column graphs on the right. These data show no effect of diet on the distance run each hour following morphine or saline. Similar to males (Fig 5) supplementary DHA did not alter total distance run in females treated with saline who increased their activity above pre-injection levels by day 8; **, ## p<0.01 vs pre-injection control of the same diet. In mice treated with morphine, total distance run decreased over time on both control and DHA diets; *,# p<0.05 vs pre-injection control of the same diet.
(DOCX)

S2 Fig n-3 PUFA supplementation does not alter cortical DHA content in female mice. Neither the chronic morphine nor the n-3 supplementation protocol altered the DHA content of the frontal cortex of female mice.
(DOCX)

S1 Table Electrophysiology parameters. Basal membrane properties and stimulating currents used to evoke EPSCs, n = 10-16/gp.
(DOCX)

S2 Table Python program for automated cell counting. Coronal slices were manually registered to the Allen Brain Atlas and then processed by this program to count the number of cells in the 64 principal brain region and exported as an excel file. Note: Paths and file names are specific to the computer used and directory structure.
(DOCX)

S3 Table The striatal connectome. The 69 primary brain regions assessed for labeling following striatal injections of the pseudorabies virus, PRV263.
(DOCX)
