# Supplementary material for: Specific behavioral and cellular adaptations induced by chronic morphine are reduced by dietary omega-3 polyunsaturated fatty acids
Source: PLoS One. 2017 Apr 5;12(4):e0175090. doi: 10.1371/journal.pone.0175090 (PMC5381919; doi:10.1371/journal.pone.0175090)
Supplement: S1 Fig — Wheel running activity was measured over 16h; 3h of light, 12h of dark, 1h of light. The distance run (cm) every hour is shown in the 3 left panels and the total distance run in the column graphs on the right. These data show no effect of diet on the distance run each hour following morphine or saline. Similar to males (Fig 5) supplementary DHA did not alter total distance run in females treated with saline who increased their activity above pre-injection levels by day 8; **, ## p<0.01 vs pre-injection control of the same diet. In mice treated with morphine, total distance run decreased over time on both control and DHA diets; *,# p<0.05 vs pre-injection control of the same diet. (DOCX) [file pone.0175090.s001.docx]

**
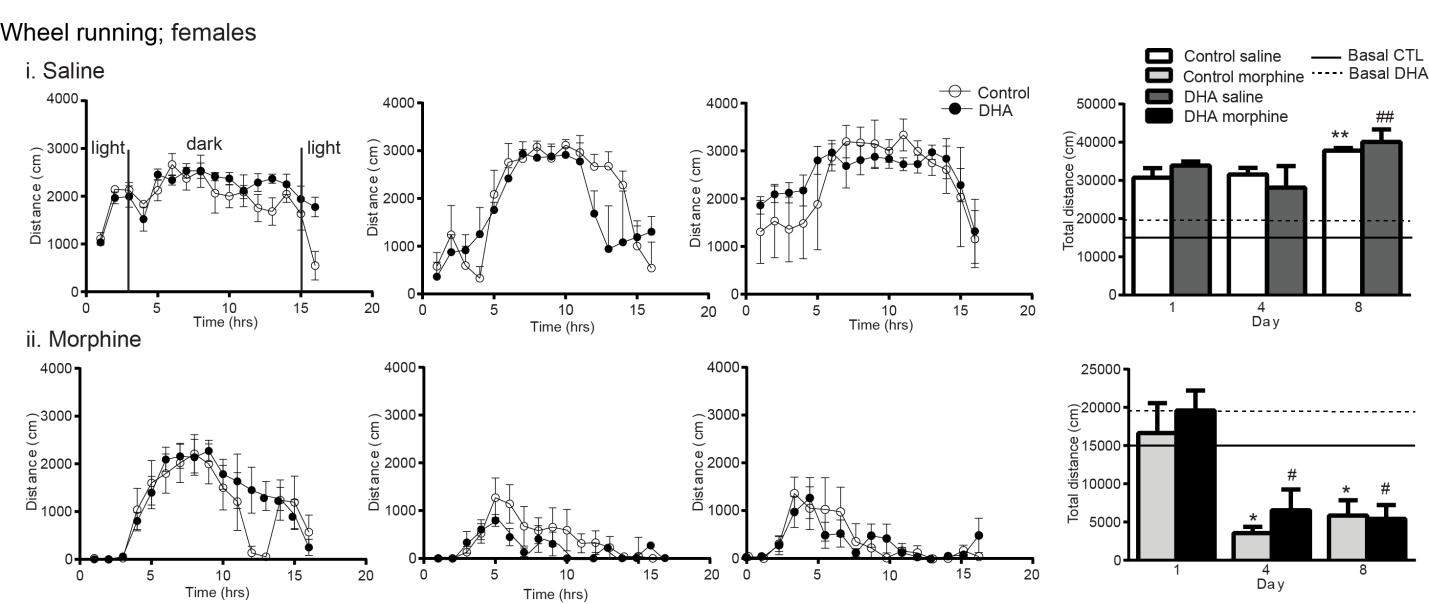
S1 Fig. n-3 PUFA supplementation does not alter wheel running in female mice.** Wheel running activity was measured over 16h; 3h of light, 12h of dark, 1h of light. The distance run (cm) every hour is shown in the 3 left panels and the total distance run in the column graphs on the right. These data show no effect of diet on the distance run each hour following morphine or saline. Similar to males (Fig 5) supplementary DHA did not alter total distance run in females treated with saline who increased their activity above pre-injection levels by day 8; **, ## p<0.01 vs pre-injection control of the same diet. In mice treated with morphine, total distance run decreased over time on both control and DHA diets; *,# p<0.05 vs pre-injection control of the same diet.
