# Supplementary material for: Specific behavioral and cellular adaptations induced by chronic morphine are reduced by dietary omega-3 polyunsaturated fatty acids
Source: PLoS One. 2017 Apr 5;12(4):e0175090. doi: 10.1371/journal.pone.0175090 (PMC5381919; doi:10.1371/journal.pone.0175090)
Supplement: S2 Fig — Neither the chronic morphine nor the n-3 supplementation protocol altered the DHA content of the frontal cortex of female mice. (DOCX) [file pone.0175090.s002.docx]

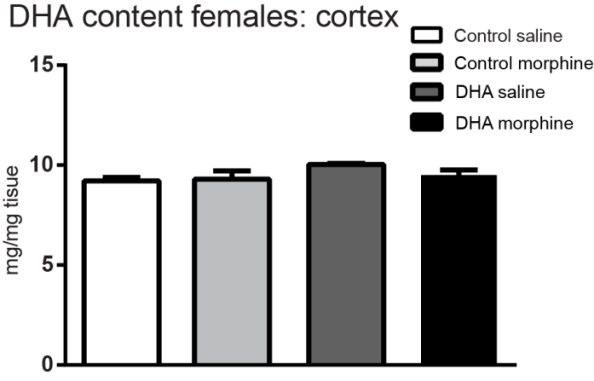
**S2 Fig. n-3 PUFA supplementation does not alter cortical DHA content in female mice.** Neither the chronic morphine nor the n-3 supplementation protocol altered the DHA content of the cortex of female mice.
