# Supplementary material for: Specific behavioral and cellular adaptations induced by chronic morphine are reduced by dietary omega-3 polyunsaturated fatty acids
Source: PLoS One. 2017 Apr 5;12(4):e0175090. doi: 10.1371/journal.pone.0175090 (PMC5381919; doi:10.1371/journal.pone.0175090)
Supplement: S1 Table — Basal membrane properties and stimulating currents used to evoke EPSCs, n = 10-16/gp. (DOCX) [file pone.0175090.s003.docx]

**S1 Table. Electrophysiology parameters:** Basal membrane properties and stimulating currents used to evoke EPSCs, n=10-16/gp

| **Group** | Membrane resistance (mΩ) | Capacitance  (pF) | Access conductance (ns) | Series resistance (mΩ) | Holding current (pA) | Stimulating current (mA) |
| --- | --- | --- | --- | --- | --- | --- |
| Control saline | 144±37 | 71±7 | 25±3 | 18±1 | -49±11 | 418±20 |
| Control morphine | 177±53 | 62±7 | 18±2 | 20±2 | -107±25 | 462±29 |
| DHA saline | 163±33 | 73±12 | 20±3 | 18±2 | -44±12 | 443±30 |
| DHA morphine | 140±36 | 67±9 | 24±3 | 15±1 | -110±47 | 486±25 |
