# Supplementary material for: Specific behavioral and cellular adaptations induced by chronic morphine are reduced by dietary omega-3 polyunsaturated fatty acids
Source: PLoS One. 2017 Apr 5;12(4):e0175090. doi: 10.1371/journal.pone.0175090 (PMC5381919; doi:10.1371/journal.pone.0175090)

**S2 Table. Python program for automated cell counting***.* Coronal slices were manually registered to the Allen Brain Atlas and then processed by this program to count the number of cells in the 64 principal brain region and exported as an excel file. Note: Paths and file names are specific to the computer used and directory structure.

**
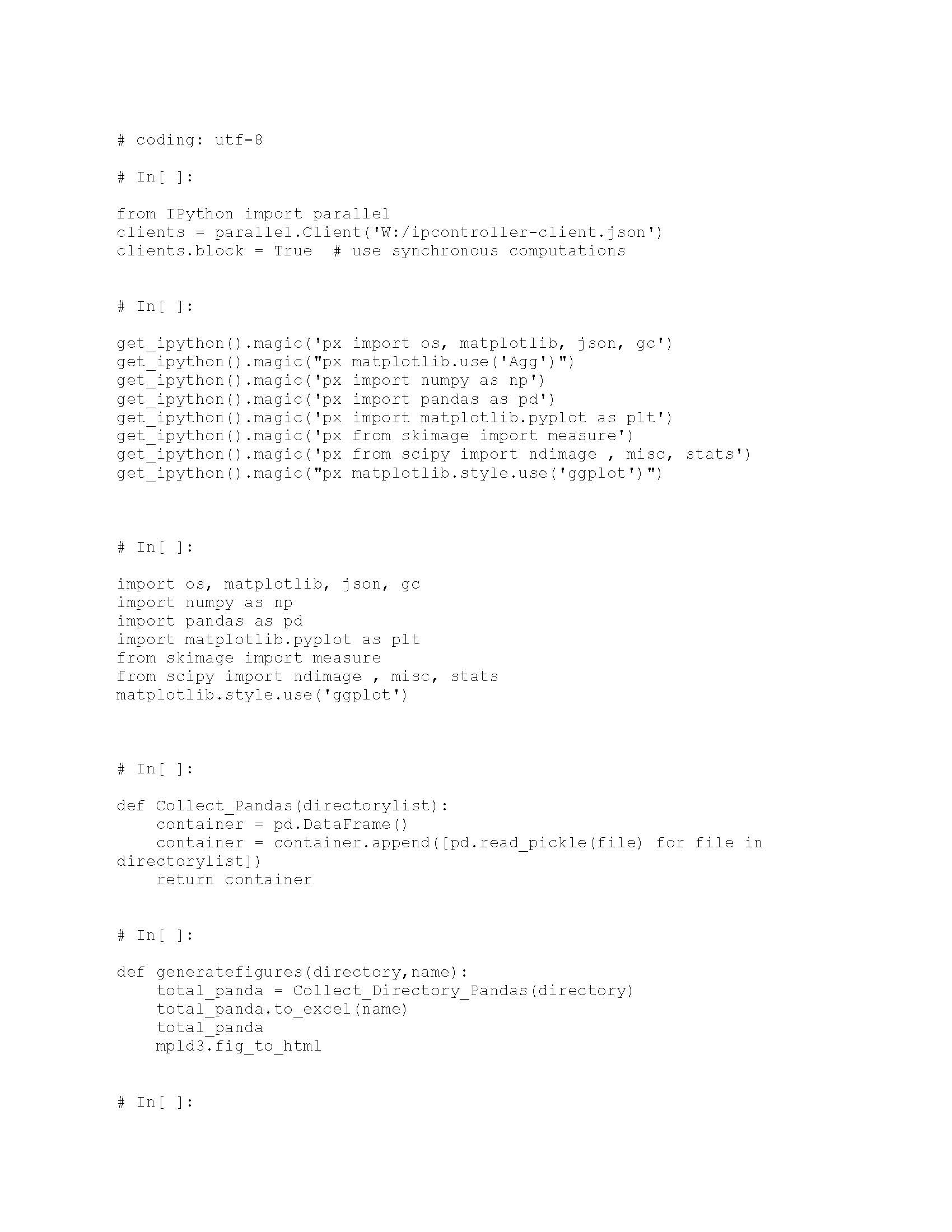
**


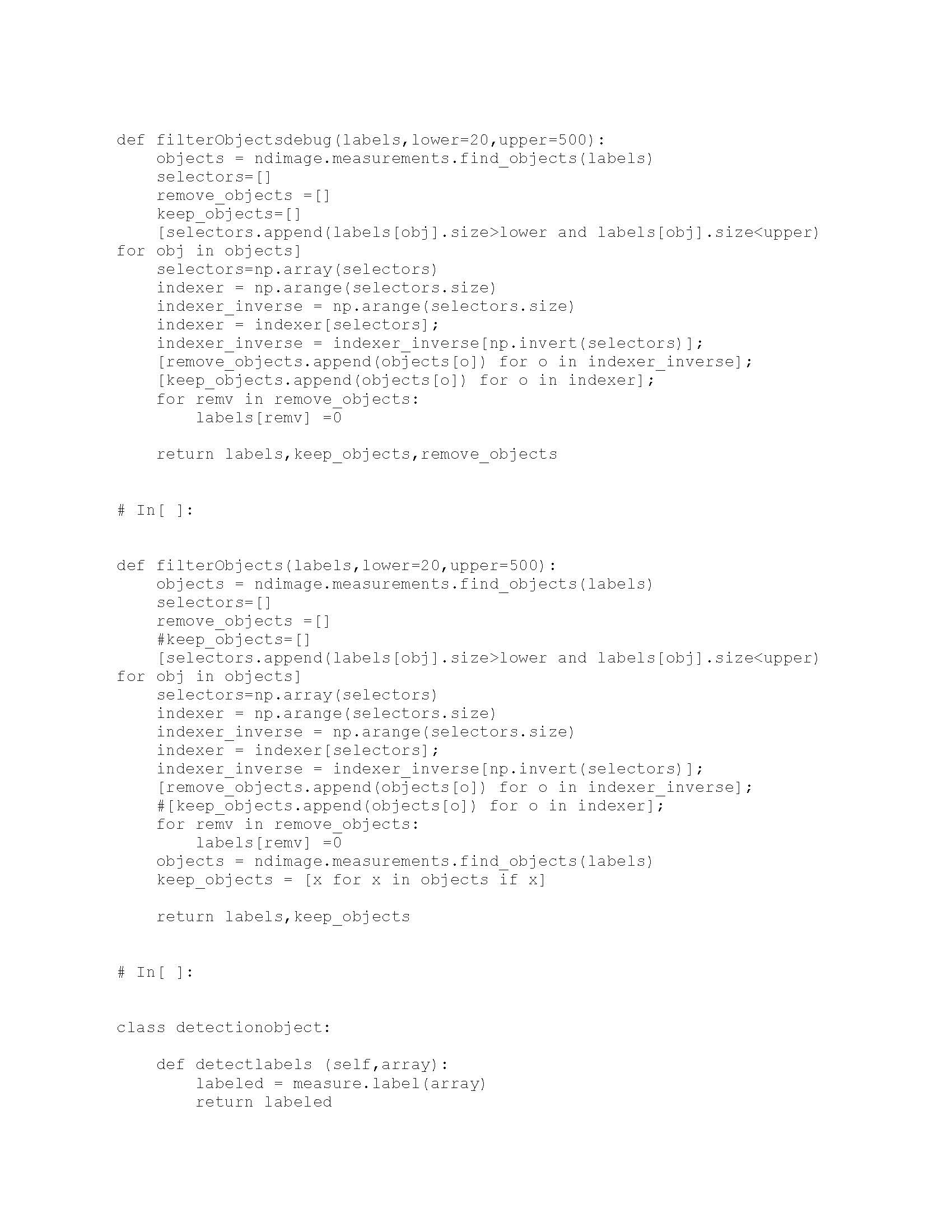


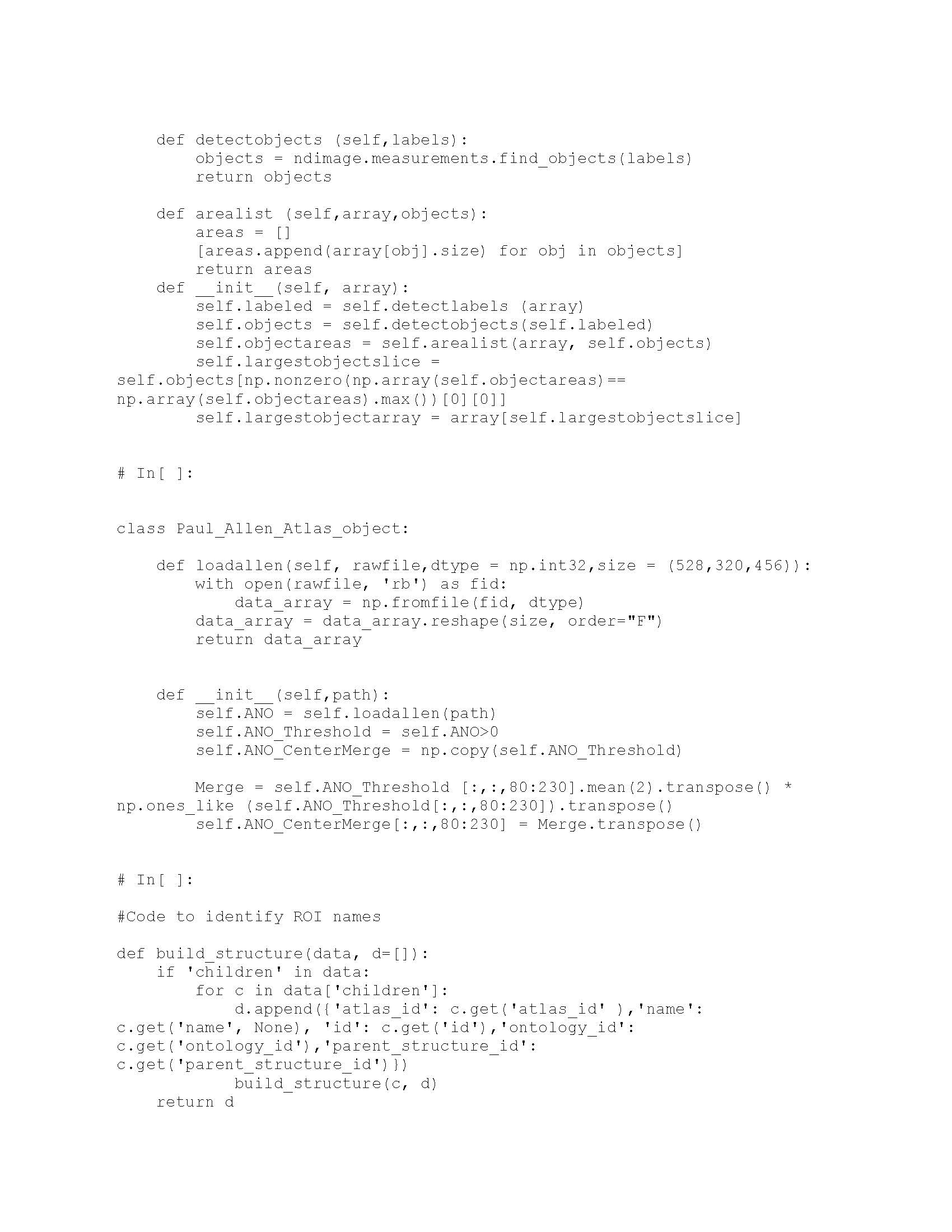


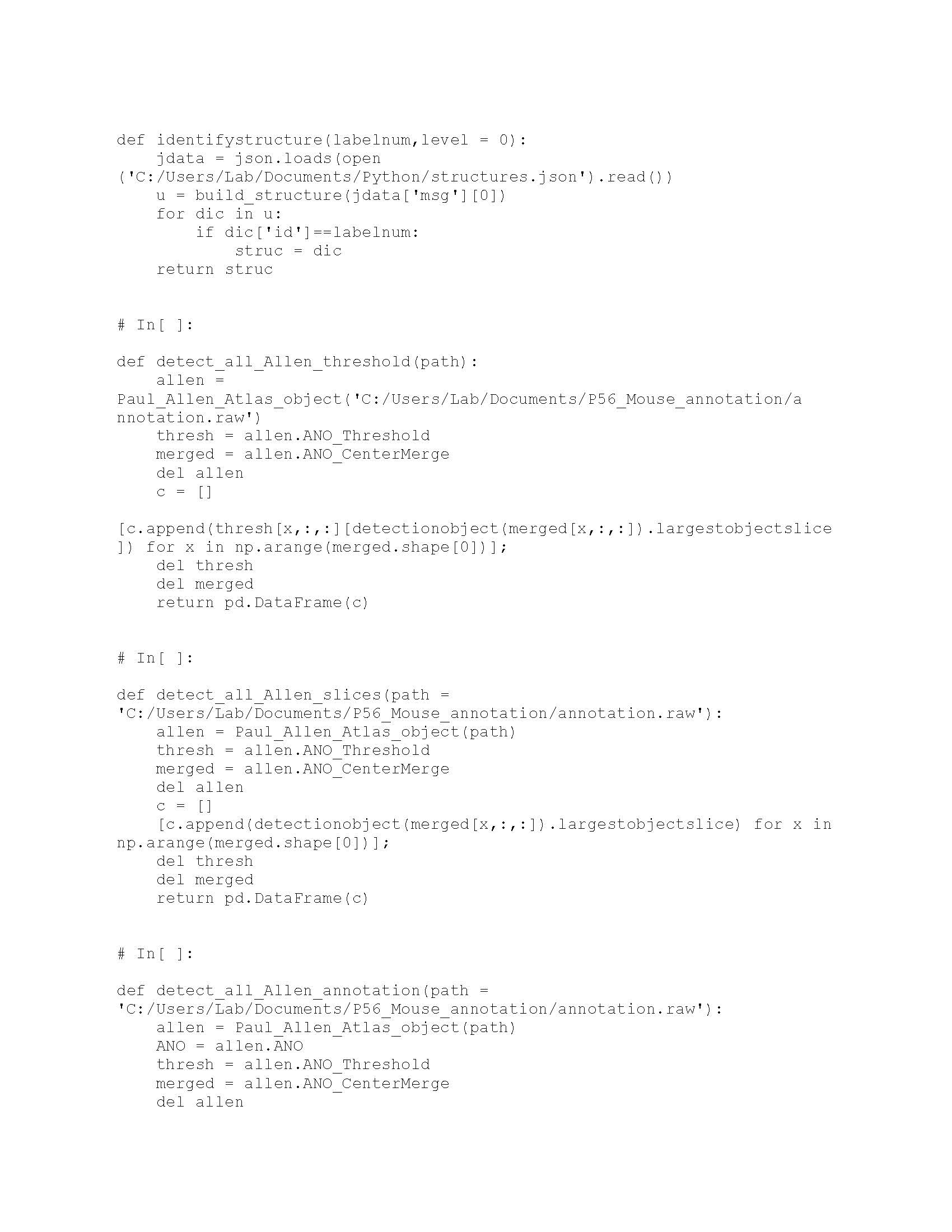


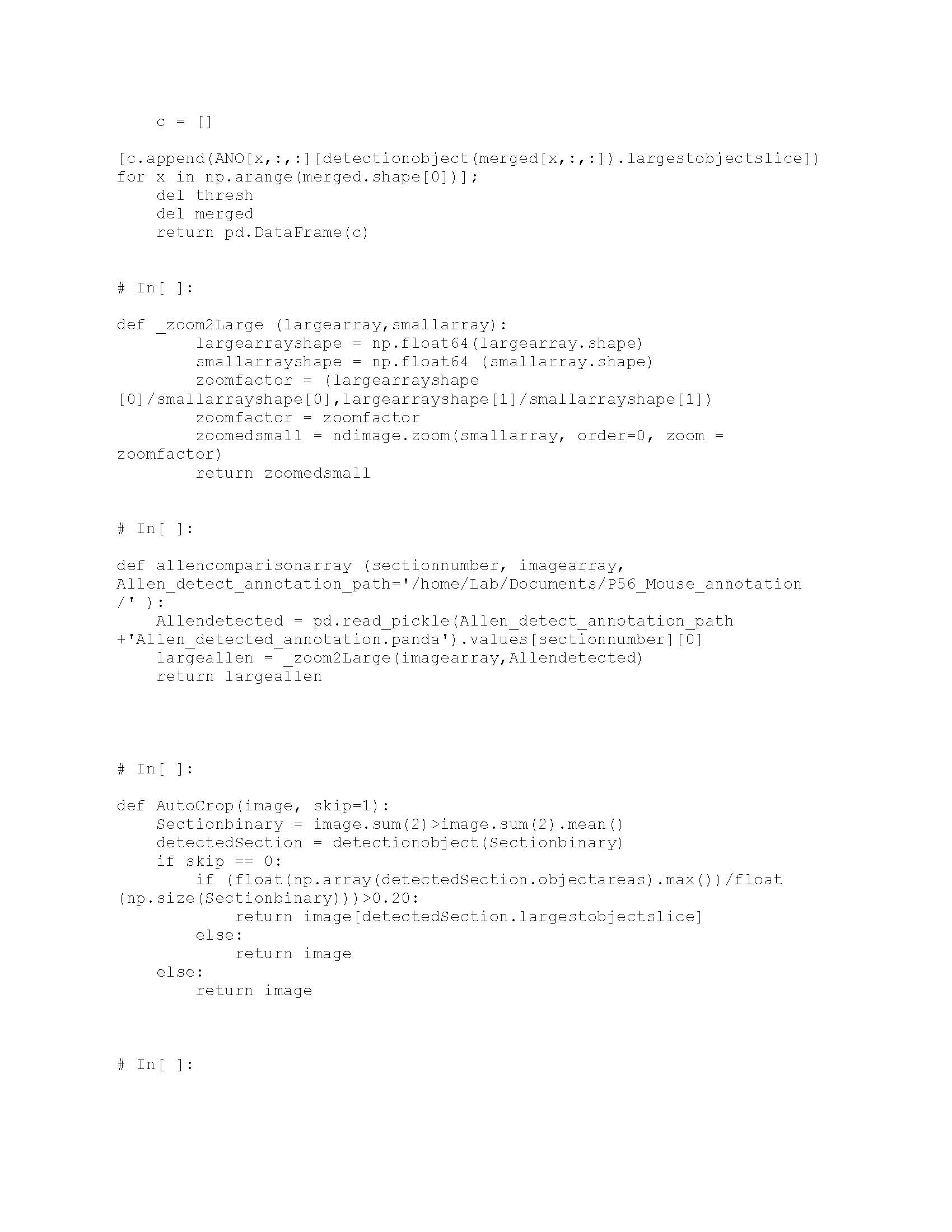


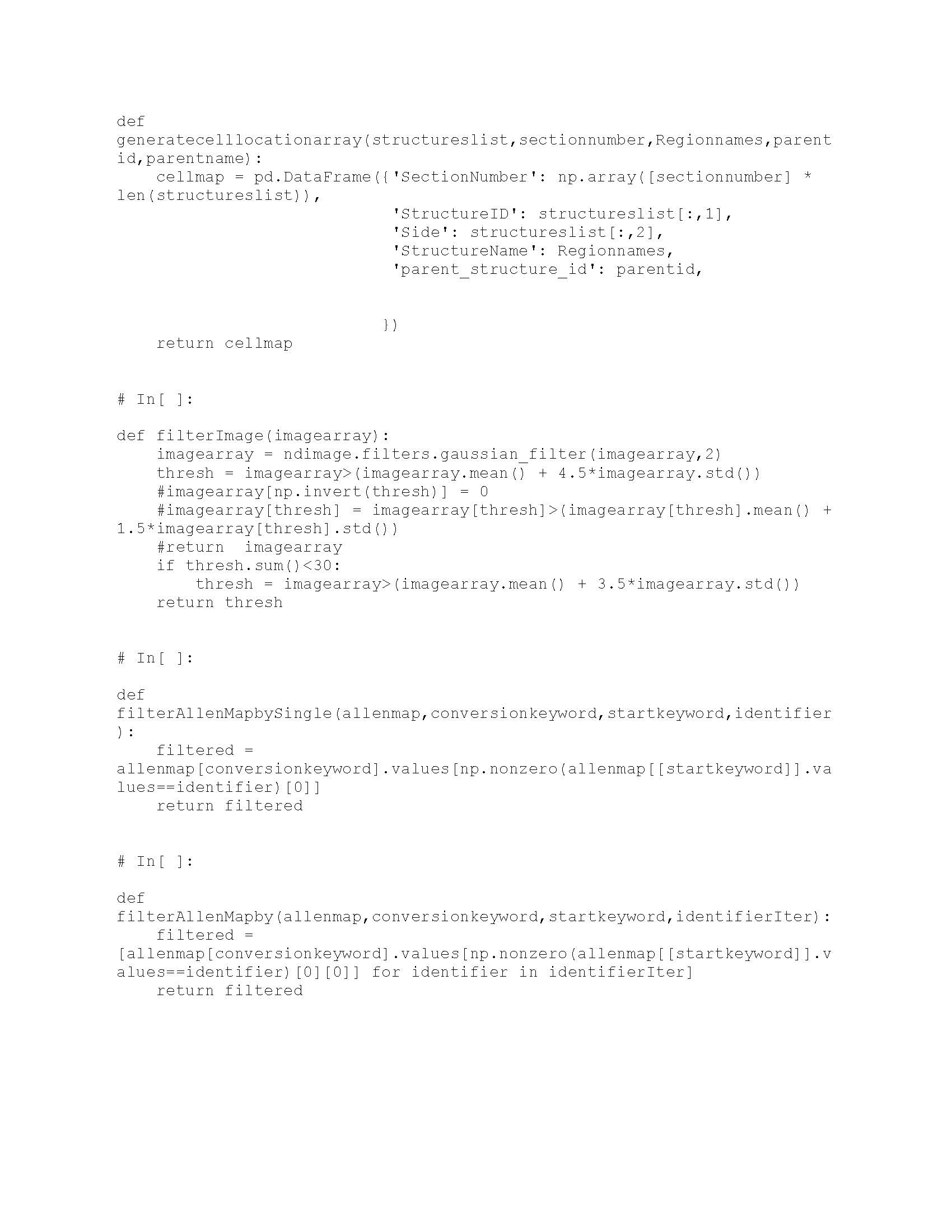


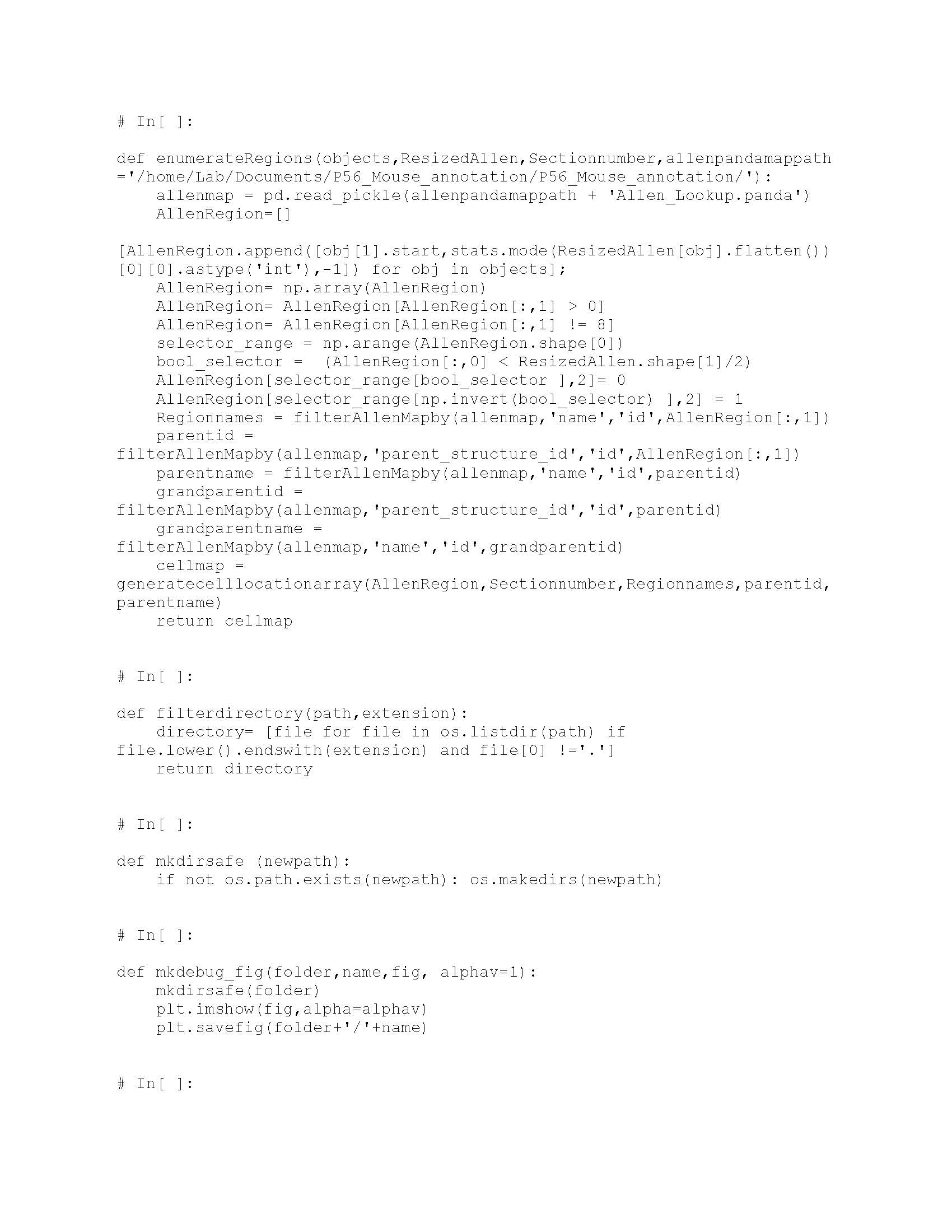


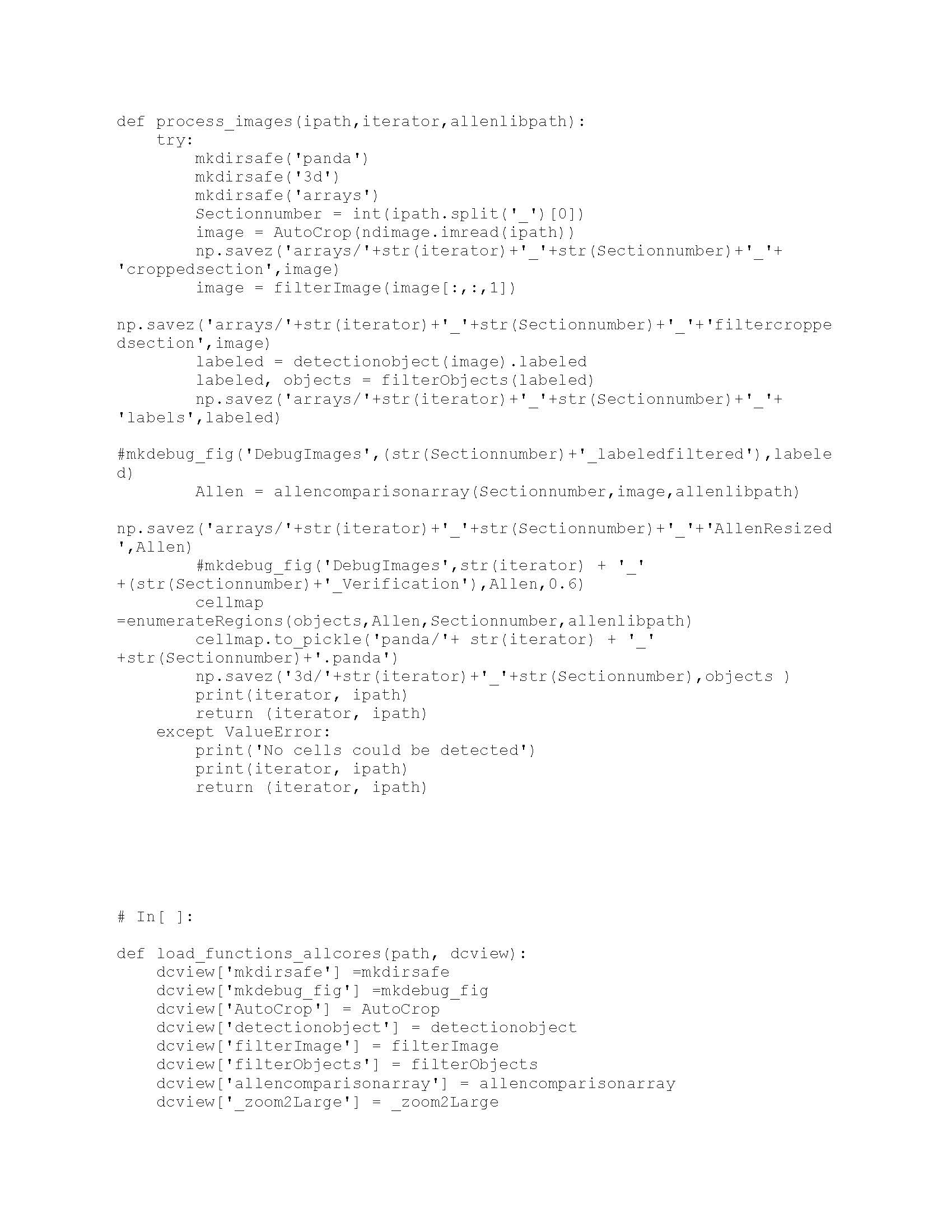


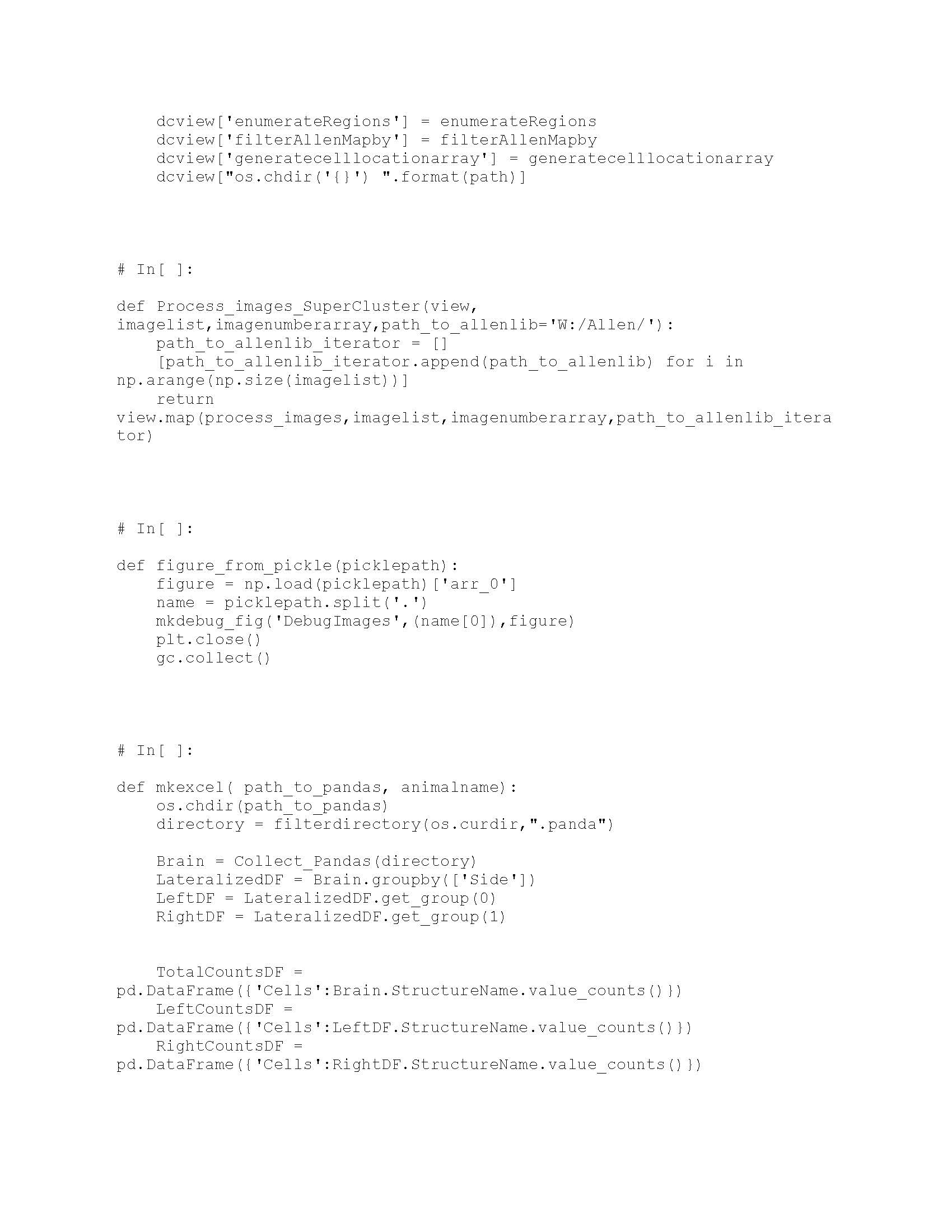


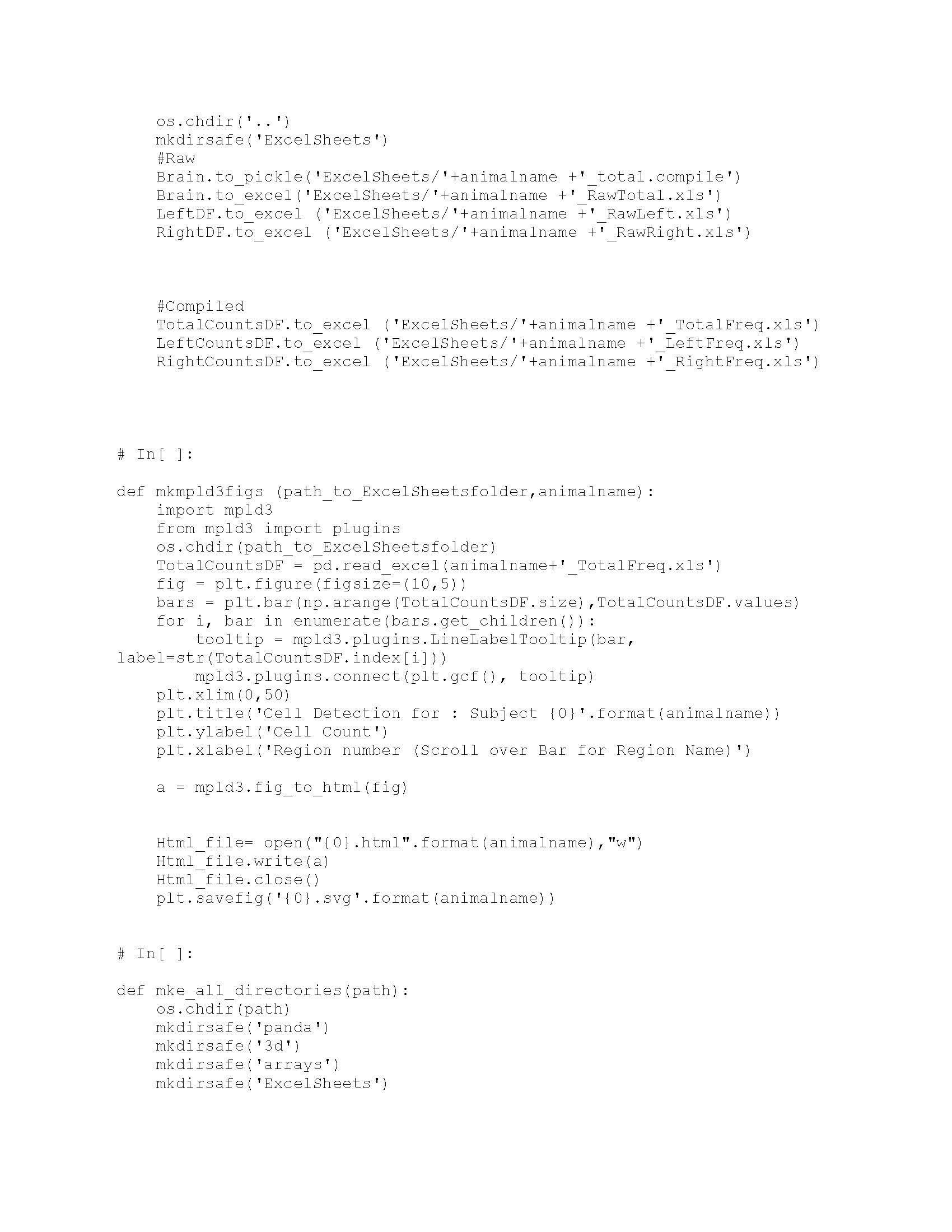


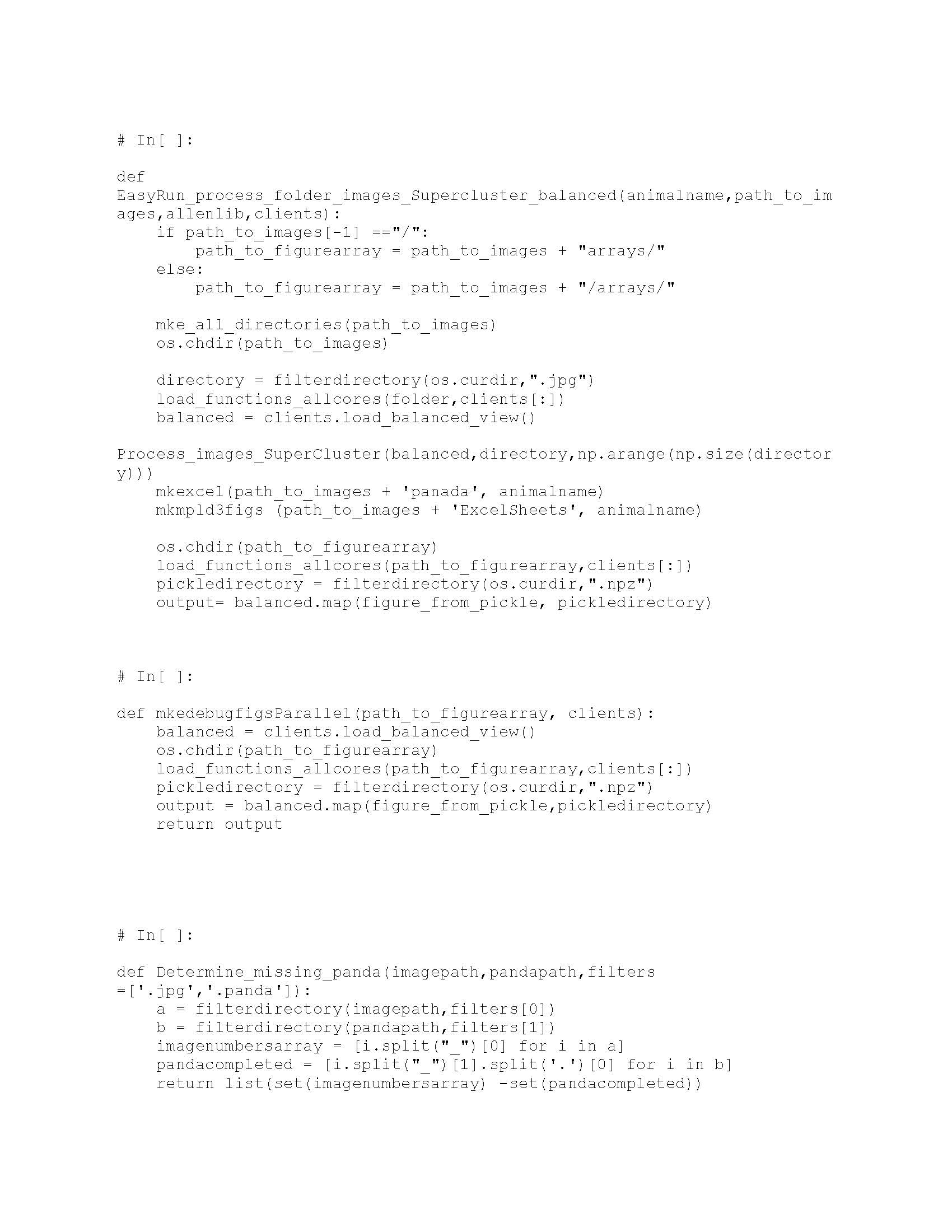


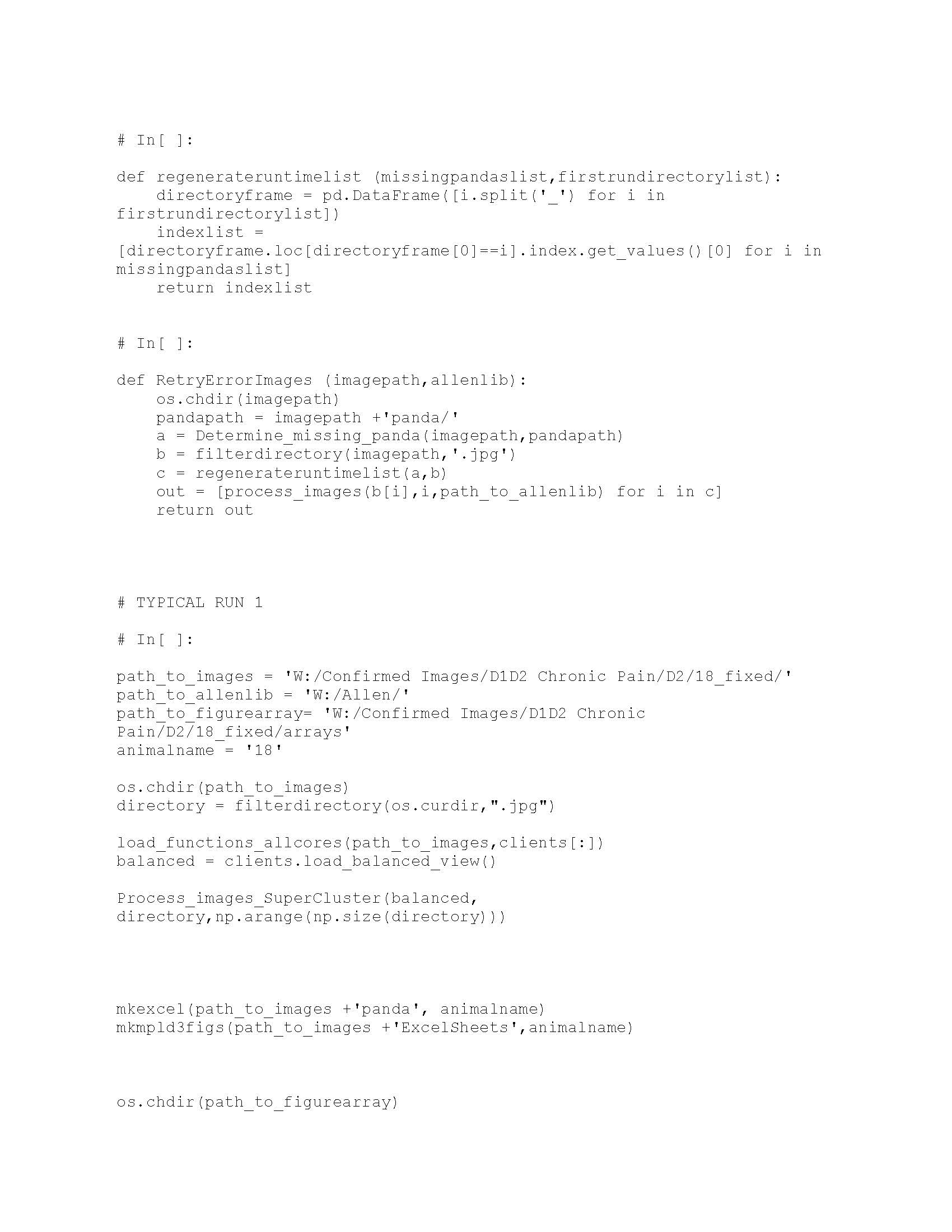


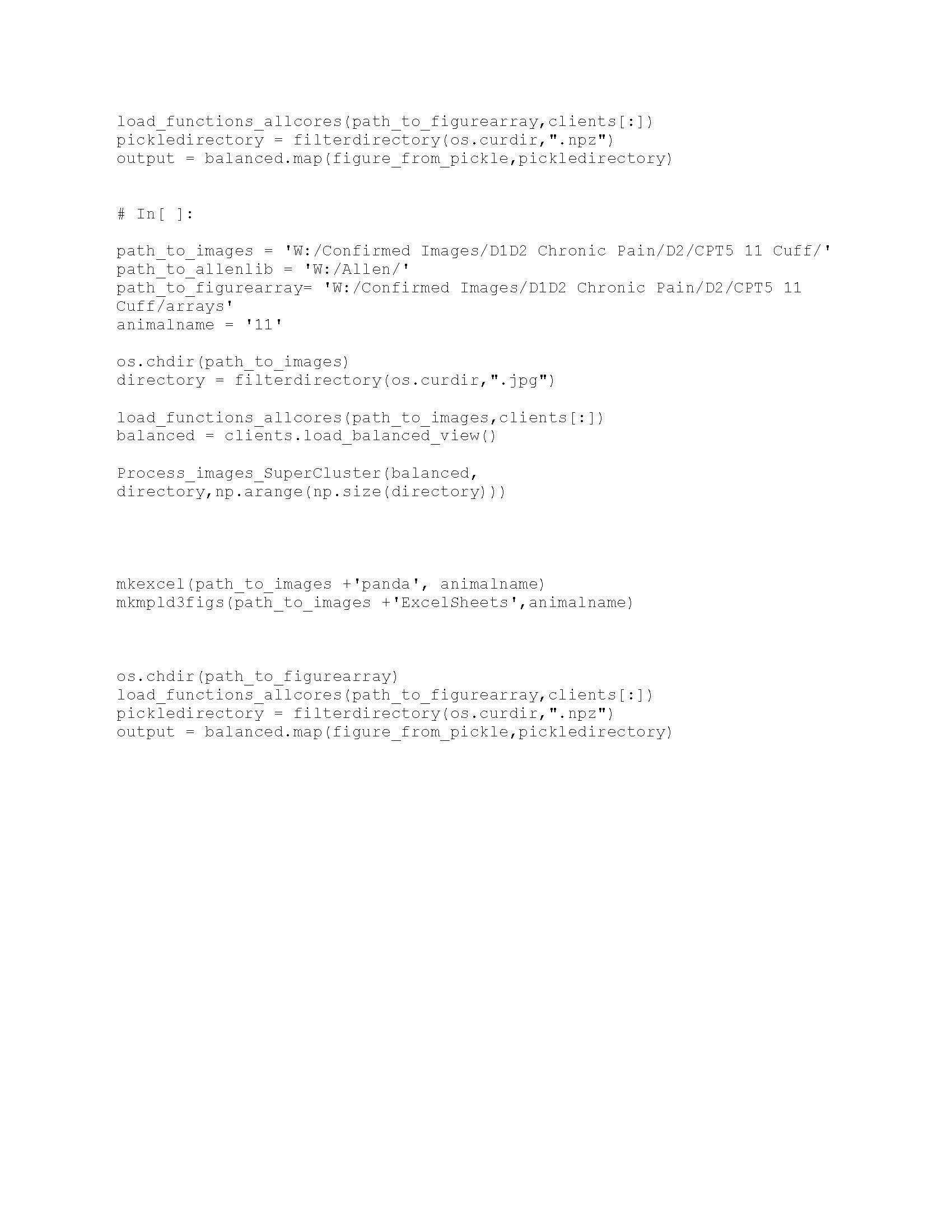

Supplement: S2 Table — Coronal slices were manually registered to the Allen Brain Atlas and then processed by this program to count the number of cells in the 64 principal brain region and exported as an excel file. Note: Paths and file names are specific to the computer used and directory structure. (DOCX) [file pone.0175090.s004.docx]
