# Supplementary material for: Specific behavioral and cellular adaptations induced by chronic morphine are reduced by dietary omega-3 polyunsaturated fatty acids
Source: PLoS One. 2017 Apr 5;12(4):e0175090. doi: 10.1371/journal.pone.0175090 (PMC5381919; doi:10.1371/journal.pone.0175090)
Supplement: S3 Table — The 69 primary brain regions assessed for labeling following striatal injections of the pseudorabies virus, PRV263. (DOCX) [file pone.0175090.s005.docx]

**S3 Table.** **The striatal connectome.** The 69 primary brain regions assessed for labeling following striatal injections of the pseudorabies virus, PRV263.

| Agranular insular area |
| --- |
| Anterior amygdalar area |
| Anterior cingulate area |
| Auditory areas |
| Basolateral amygdalar nucleus |
| Basomedial amygdalar nucleus |
| Bed nucleus of the accessory olfactory tract |
| Caudoputamen |
| Central amygdalar nucleus |
| Cerebellum |
| Cerebrum |
| Claustrum |
| Cuneiform nucleus |
| Dentate gyrus |
| Ectorhinal area |
| Edinger-Westphal nucleus |
| Endopiriform nucleus |
| Entorhinal area |
| Fasciola cinerea |
| Field CA1 |
| Field CA2 |
| Field CA3 |
| Fundus of striatum |
| Gustatory areas |
| Hypothalamus |
| Induseum griseum |
| Infralimbic area |
| Intercalated amygdalar nucleus |
| Lateral amygdalar nucleus |
| Lateral septal complex |
| Medial amygdalar nucleus |
| Medulla |
| Midbrain |
| Midbrain raphe nuclei |
| Midbrain reticular nucleus |
| Midbrain reticular nucleus, retrorubral area |
| Midbrain, sensory related |
| Nucleus accumbens |
| Oculomotor nucleus |
| Olfactory areas |
| Olfactory tubercle |
| Orbital area |
| Pallidum |
| Parasubiculum |
| Pedunculopontine nucleus |
| Periaqueductal gray |
| Perirhinal area |
| Pons |
| Posterior amygdalar nucleus |
| Posterior parietal association areas |
| Postsubiculum |
| Prelimbic area |
| Presubiculum |
| Pretectal region |
| Primary motor area |
| Primary somatosensory area |
| Red nucleus |
| Retrosplenial area |
| Secondary motor area |
| Subiculum |
| Substantia nigra, compact part |
| Substantia nigra, reticular part |
| Superior colliculus, motor related |
| Supplemental somatosensory area |
| Temporal association areas |
| Thalamus |
| Ventral tegmental area |
| Visceral area |
| Visual areas |
